# Cesarean section deliveries in the occupied Palestinian territory (oPt): An analysis of the 2006 Palestinian Family Health Survey

**DOI:** 10.1016/j.healthpol.2009.07.006

**Published:** 2009-12

**Authors:** Hanan F. Abdul-Rahim, Niveen “Mohammad Elias” Abu-Rmeileh, Laura Wick

**Affiliations:** a International Affairs Program, Qatar University, P.O. Box 2713, Doha, Qatar; b Institute of Community and Public Health, Birzeit University, P.O. Box 154, West Bank, oPt

**Keywords:** Cesarean sections, Prevalence, Developing countries, Health systems

## Abstract

**Objective:**

Against the backdrop of a rise in cesarean section deliveries from 6.0% in 1996 to 14.8% in 2006, the objective of this study was to investigate socio-demographic, clinical and service-related factors associated with cesarean sections in the occupied Palestinian territory.

**Methods:**

Data from the Palestinian Family Health Survey 2006 were used to examine last births in the 5 years preceding the survey to women aged 15–49 years. Bivariate and multivariate associations between type of delivery (dependent variable) and selected factors were analyzed using logistic regression. Selected maternal outcomes were also investigated with *type of delivery* as the independent variable.

**Results:**

Cesarean section deliveries were significantly associated with maternal age (35+ years), primiparity, low birth weight and residence area in the West Bank and Gaza. There was no significant difference in the prevalence of cesarean deliveries by sector in the West Bank, but in Gaza, they were significantly more common in the governmental sector.

**Conclusions:**

There is a need for detailed audits of cesarean section deliveries, nationally and at the facility level, in order to avoid unnecessary interventions in the context of high fertility, rising poverty and fragmented health services. Variations by governorate should be studied further for focused interventions.

## 1 Introduction

The prevalence of cesarean section deliveries continues to rise in most developed [1] and developing [2] countries. Although the reasons for the global increase beyond the WHO recommended rate of 10–15% might have similarities across regions [3], some attributing factors may take on more importance than others in specific contexts. Variations in the prevalence of cesarean section deliveries may also exist within the same country, reflecting contradictions of unmet need in some circumstances and unnecessary intervention in others [4]. The implications of this trend may be more harmful for the health of women and newborns in developing countries, where high fertility and substandard health care may increase the short and long-term risks of cesarean deliveries [5] and where limited resources can be diverted from other necessary and effective interventions [6].

In several Middle Eastern countries, the frequency of cesarean section deliveries is on the rise [2]. However, there are variations from a low of 1.4% in Yemen to a high of 16.0% in Bahrain [7]. In the occupied Palestinian territory (oPt), the prevalence has more than doubled from 6.0% in 1996 to 14.8% in 2006 [8,9]. Despite this increase, there have not been studies to investigate the determinants of cesarean section deliveries in the complex Palestinian context. Such a study would be particularly relevant, as medicalized childbirth procedures are frequently being adopted in a context of rising poverty, disrupted health services, scarce human and financial resources, and political instability [10]. Though most women in the oPt give birth in health facilities with a high rate of skilled attendance, access to hospitals can often be unpredictable due to over 600 military checkpoints [11] restricting mobility. Thus, investigating the factors associated with cesarean section deliveries is necessary to inform decision-makers about how to develop context-specific strategies to reduce unnecessary operations and avoid unjustified and costly interventions [12].

This study investigates the determinants of cesarean section deliveries in the oPt based on the self-reporting of a representative sample of women of reproductive age in the West Bank and Gaza Strip in the Palestinian Family Health Survey 2006. The study will examine the association of maternal socio-demographic characteristics, service-related factors, and selected clinical factors with the type of delivery (cesarean vs. vaginal births). Selected maternal outcomes will also be compared by type of delivery.

## 2 Materials and methods

Analysis was based on data from the Palestinian Family Health Survey (PFHS) conducted by the Palestinian Central Bureau of Statistics (PCBS) in 2006 in cooperation with the Pan-Arab Project for Family Health (PAPFAM), UNICEF, and UNFPA. The PFHS is part of a regional survey supervised by the League of Arab States and conducted in several Arab countries, and it is designed to collect demographic and health data pertaining to the Palestinian population living in the occupied Palestinian territory, with a focus on demography, fertility, family planning and maternal and child health, in addition to issues of the youth and elderly [9].

The survey used a stratified two-stage random sampling design and included four questionnaires to ensure comparability with the previous health surveys whereas also including the core indicators of the Multiple Indicator Cluster Survey (MICS). The latter is supervised by UNICEF and designed to fill gaps in data for monitoring the socioeconomic and health situation of women and children^1^. The first questionnaire covered household health and MICS indicators; the second questionnaire (which is a part of the first) covered MICS indicators only, and the third and fourth questionnaires pertained to unmarried youth and the elderly, respectively. For the purposes of this analysis, only the first and second questionnaires were used, which were collected from 7056 and 6182 non-overlapping households, respectively. Both questionnaires included sections on household members, ever-married women aged below 55 years, and children below 5 years. Of the 13,238 households in the sample, 11,661 were interviewed at a response rate of 88.0%, and a total of 10,648 ever-married women (aged 15–54 years) were interviewed, at a response rate of 98.3% [9].

Data were analyzed using the Statistical Package for the Social Sciences (SPSS) version 15.0. Analysis was restricted to women aged 15–49 years old who had at least one live birth in the 5 years preceding the survey, focusing on their last live birth. Nine cases were excluded from the analysis as they were of cesarean section deliveries which were reported to have taken place in settings without surgical facilities, such as physicians’ clinics [13].

Bivariate and multivariate analyses were conducted with *type of delivery* (CS/vaginal) as the dependent variable using logistic regression. The *vaginal delivery* category included both non-instrumental and instrumental (forceps or vacuum) deliveries, as the frequency of the latter was only 2.7%. On the basis of the results of the bivariate analysis, two separate multivariate models were used for the West Bank and Gaza Strip. Potential interactions between maternal age and parity were tested and reported in both models.

In the West Bank, 11 governorates were recoded into northern (6 governorates), central (3 governorates) and southern (2 governorates) areas, whereas in the Gaza Strip, 5 districts were also grouped into northern (1 governorate), central (2 governorates), and southern (2 governorates) areas. Delayed access to delivery facilities due to Israeli military procedures was considered. An *access indicator* was constructed by counting the positive responses of women to four questions on encountering *delays at a military checkpoint*, *complete closure of a military checkpoint*, *restricted mobility due to the Separation Wall*, or difficulty due to *curfew and closure*. However, in the multivariate analysis, the access variable was only used in the West Bank model. The Gaza Strip is characterized by strict closure of the borders with Israel and thus the West Bank, which is non-contiguous, and Egypt, but movement within the Strip is relatively unimpeded by checkpoints.

To assess selected outcomes of cesarean section vs. normal deliveries, initiation of breastfeeding, mean breastfeeding duration, and the proportion of women who visited a physician (general practitioner or specialist) in the postpartum period were compared with *type of delivery* as the independent variable. The independent sample t-test was used to assess the significance of the association between duration of breastfeeding and type of delivery, whereas the Chi-square test was used for assessing associations with categorical variables.

## 3 Results

Characteristics of births to women in the oPt reporting one or more live births in the 5 years preceding the survey are presented in Table 1. The prevalence of cesarean section deliveries in the oPt was 14.8%, with no difference between the West Bank and Gaza Strip (15.2% and 14.3%, respectively). Similarly, there were no significant differences in the prevalence of instrumental deliveries (2.7% in the West Bank and 2.6% in the Gaza Strip). Variations in the prevalence of cesarean section levels by governorate ranged from 10.7% to 22.3% in the West Bank and from 12.1% to 18.9% in the Gaza Strip (data not shown).

The majority of births in the oPt are facility-based, and almost all are attended by skilled personnel (97.1%). Multiparity is common, given the high total fertility rate of 4.5. Antenatal care is almost universal (98.8%), but less than one third of women who had delivered within 6 weeks received postnatal care (29.5%) [9]. Over one half of all deliveries occur in the governmental (public) sector. However, there are significant regional differences, with 72.6% of deliveries in Gaza occurring in governmental hospitals compared with 47.3% in the West Bank. As discussed earlier, the questions on access to place of delivery were more relevant to the political situation in the West Bank, which is characterized by checkpoints and closures between areas, and much less applicable to Gaza, where the access difficulties are of a different nature (external border closure rather than closures *within* Gaza areas). As a result, 14.9% of women in the West Bank reported access difficulties compared with only 0.6% in the Gaza Strip.

Determinants of cesarean section deliveries are investigated in Table 2 using bivariate and multivariate analyses. In the West Bank, older age (35+ years), primiparity, low birth weight and area of residence (north and center) were significantly associated with cesarean section deliveries in multivariate analysis. Reported difficulty in accessing a hospital lost statistical significance when adjusted for several factors, including area. No significant differences were found between the private and governmental sectors in the prevalence of cesarean section deliveries.

In the Gaza Strip, similar associations were seen. Cesarean section deliveries were significantly associated with higher maternal age, primiparity, low birth weight and area of residence (north). However, unlike the West Bank, cesarean sections were significantly more common in the governmental sector than in the private sector. In both regions, the associations of parity and maternal age were stronger in the multivariate analysis, so an age-parity interaction term was included. However, for both regions, it must be noted that birth weights are for pre-term and full-term babies together, as information on gestational age is not available.

Selected maternal and newborn outcomes related to cesarean deliveries are shown in Table 3. Breastfeeding rates were significantly higher among women who had delivered vaginally (*p* < 0.001), but the duration of breastfeeding was not significantly different between the two groups. Predictably, a higher proportion of women who had delivered by cesarean section reported visiting a physician post-partum, probably because a cesarean section delivery is considered a surgical procedure requiring a post-operative examination. Women were asked about symptoms of complications during and immediately after delivery as well as during the first 6 weeks following delivery. However, the number of women who reported such symptoms in either time period was too small to allow for reliable comparisons.

## 4 Discussion

The trend of rising cesarean sections observed in the developed and developing world is also evident in the oPt, where the prevalence of cesarean section deliveries has more than doubled over the past decade [9]. Although the current level of 14.8% may not be as alarming as that found in some other countries [7,14,15]**,** the trend raises issues concerning the quality of childbirth care in the context of limited resources, fragmented services and high fertility. It draws attention to the need to explore the factors that may explain this trend for health and policy implications.

In general, population-based surveys, such as the Palestinian Family Health Survey, are an adequate source of information on the prevalence of cesarean sections for the purposes of national and global monitoring [4], particularly in settings such as this where data from health facilities is either lacking or of poor quality. The Palestinian health surveys since 1996 and until 2006 have had very high response rates. Furthermore, the use of standardized instruments with strict fieldworker training protocols and quality control checks on data entry and processing ensure that the information collected is comparable over the different surveys.

Consistent with other studies, cesarean deliveries in the West Bank and in Gaza were more common among older and primiparous women [16]. The interaction between age and parity meant that older primiparous women were more likely to be delivered by a cesarean section than vaginally compared with younger women with previous deliveries. Women in the oPt tend to marry and start childbirth early in their lives [9], so having a first birth at an older age is rather unusual in this context. It is possible that physicians treat older primiparous women with more caution and consider their pregnancies “precious”, or that they do indeed have more complications. On the other hand, education and wealth were not significant factors, and neither was the sector (public or private) in which the deliveries took place.

Low birth weight (full-term *and* pre-term) babies were more likely to have been delivered by cesarean sections, as shown in some other studies in both developed [17] and developing [18,19] countries. However due to the data limitations in this cross-sectional survey it is not possible to determine whether the low birth weight was due to cesarean sections performed prematurely [20] or whether cesarean sections were chosen deliberately for low birth weight infants.

Area of residence had a significant association with type of delivery, although it was independent of access difficulties as had been expected. Furthermore, there were substantial variations in the prevalence of cesarean sections at the governorate level, although the number of deliveries in the sample did not allow for this variable to be used in multivariate analysis. This variation by area and governorate may suggest area-specific factors in clinical services which need to be standardized. Although national evidence-based guidelines to standardize obstetric care have been established and disseminated [21], there is no evidence yet of their implementation. These variations should be studied for targeted and context-appropriate interventions.

The absence of an association between sector of delivery and cesarean sections in the West Bank was contrary to the international trend, where cesarean births are usually associated with the private sector due to the profit motive [22,23]. However, rising poverty in the oPt has rendered the private sector inaccessible to most women. Furthermore, the *Al Aqsa* insurance, which was instituted with the beginning of the second Uprising (*Intifada*), made maternity services, including cesarean deliveries, free of charge in the governmental sector. Anecdotal evidence suggests that some women who go to the private sector for delivery often transfer to the governmental hospitals *during* labor, if the need for an emergency cesarean section becomes apparent, to avoid the high cost of an operation. Moreover, obstetricians in the governmental hospitals frequently practice also in private facilities to supplement their low salaries [24], where the issue of timing their presence in different institutions becomes paramount. The need to program deliveries has been shown in other countries to be a reason behind rising interventions [22].

Alternatively in the Gaza Strip, cesarean deliveries were actually lower in the private sector. The higher cesarean section prevalence in the governmental hospitals is most likely due to a multiplicity of factors linked to issues of financial and geographic access resulting from poverty and on-going conflict as well as poor quality of care, where overcrowding and under-staffing do not permit support during labor [25].

Education is believed to influence cesarean section rates either through age and parity, with more educated women delaying childbirth [26], or through social factors, where educated women perceive cesarean deliveries as “the optimum form of delivery” [27]. In the absence of reliable information on elective cesareans (and on indications for cesarean sections in general) one might speculate that, in the Palestinian case, the demand for CS may not be influenced by the “westernization” which is often assumed to accompany higher education levels [28], as much as by other contextual factors that could not be fully captured in the surveys, namely the unstable political situation and the fear of closures, which generally disrupt the organization of effective services and could cause women and providers to wish to control the time of birth.

The short term consequences of a cesarean delivery are especially important in this context. Data on outcomes of maternal and neonatal morbidity are not available and are needed to improve quality of care. However, consistent with other studies, cesarean deliveries in this survey were associated with lower breastfeeding rates most likely due to difficulty in initiating breastfeeding [29], especially since cesarean sections are mostly conducted under general anesthesia [30]. In other studies, delayed first contact between a mother and her baby has been shown to affect the maternal emotional well being in the postpartum [31,32].

## 5 Conclusion

Some determinants of cesarean delivery in the oPt such as primiparity, older age and area of residence were similar to what might be found in other parts of the world. This similarity might in itself raise questions regarding criteria for clinical decision-making in a context of on-going poor access, high fertility and scarce resources. In particular the wide use of cesarean delivery for a first birth should be carefully audited under such conditions. The differences in the levels of cesarean section deliveries by area of residence and by governorates indicate that there might be context-specific factors, independent of access difficulties that require more in-depth analysis for appropriate intervention planning. Improved quality of care including adequate reporting on indications and outcomes of cesarean delivery [33] is needed to audit practices and identify the gaps in appropriate obstetric care. Given the financial and human resource constraints for health care in the oPt [34] and the desire for large families, identifying and reducing cesarean sections for non-medical reasons should be a major priority for politicians, policy-makers and providers.

## Figures and Tables

**Table 1:** Sample characteristics of women in the oPt reporting one or more live births in the 5 years preceding the survey (2001–2005) and of the last birth.

Characteristics	Births (%)
	*N* = 6113
Mother's age (years)	
15–19	176 (2.9)
20–34	4254 (69.6)
35+	1683 (27.5)

Highest educational attainment	
None	424 (6.9)
Primary	1259 (20.6)
Preparatory	2245 (36.7)
Secondary	1353 (22.1)
Above secondary	832 (13.6)

Parity	
1	557 (9.1)
2–4	2797 (45.8)
5+	2759 (45.1)
Mean ± SD	4.53 ± 2.51
Median	4.00

Region of residence	
West Bank	3590 (58.7)
Gaza Strip	2523 (41.3)

Residence locality	
Urban	3257 (53.3)
Rural	1770 (29.0)
Refugee Camp	1086 (17.8)

Type of delivery	
Normal (vaginal, non-instrumental)	5034 (82.5)
Instrumental (vacuum and/or forceps)	164 (2.7)
Cesarean section	903 (14.8)

Place of delivery	
Government hospital or center	3521 (57.6)
Private hospital or center	1446 (23.7)
UNRWA hospital or center	193 (3.2)
NGO hospital or center	377 (6.2)
Private doctor's clinic	284 (4.6)
Home	177 (2.9)
Other*	112 (1.8)
Reported one or more difficulties in reaching place of delivery†	546 (8.9)

* The “other” category includes deliveries in maternity homes, at checkpoints, on the road, or in Israeli hospitals.

† On the basis of a positive response to one or more of four questions asking about delays at a military checkpoint, complete closure of a military checkpoint, restricted mobility due to the Wall, or difficulty due to curfew and closure.

**Table 2:** Cesarean section deliveries raw and adjusted for selected background characteristics in the West Bank and Gaza.

Characteristic	West Bank			Gaza Strip		
	*N* = 3584			*N* = 2517		
	% CS	Crude OR	Adjusted OR*	% CS	Crude OR	Adjusted OR*
		(95% CI)	(95% CI)		(95% CI)	(95% CI)
Maternal age (yr)						
15–34	12.2	Ref	Ref	12.1	Ref	Ref
35+	23.2	2.17 (1.80–2.62)	2.50 (1.94–3.21)	19.9	1.81 (1.43–2.28)	2.13 (1.56–2.89)

Parity						
1	22.5	2.03 (1.50–2.76)	2.22 (1.58 – 3.14)	20.5	1.79 (1.26–2.56)	2.46 (1.60–3.78)
2–4	12.5	Ref	Ref	12.6	Ref	Ref
5+	16.9	1.42 (1.17 – 1.73)	0.93 (0.73–1.20)	14.3	1.16 (0.91–1.48)	0.59–1.12

Maternal educational attainment						
<Secondary	15.3	Ref		15.2	Ref	
≥Secondary	14.8	0.96 (0.79–1.17)		13.0	0.83 (0.66–1.05)	

Wealth						
Poorest fifth	14.9	Ref		14.0	Ref	
Wealthiest fifth	17.3	1.20 (0.89–1.60)		15.9	1.16 (0.81–1.68)	

Residence locality						
Urban	15.4	Ref		14.6	Ref	
Rural	15.1	0.98 (0.81–1.18)		15.6	1.08 (0.71–1.65)	
Refugee camp	14.8	0.96 (0.69–1.33)		13.3	0.90 (0.70–1.16)	

Area of residence						
North	16.4	1.34 (1.08–1.67)	1.44 (1.13–1.84)	18.9	1.65 (1.22–2.22)	2.12 (1.57–3.16)
Center	17.9	1.49 (1.14–1.94)	1.49 (1.12–1.98)	13.3	1.08 (0.82–1.42)	1.29 (0.95–1.73)
South	12.8	Ref	Ref	12.4	Ref	Ref

Access to place of delivery						
No difficulties	14.4	Ref	Ref			
Reported difficulties	19.4	1.43 (1.13 – 1.80)	1.19 (0.92–1.54)			

Sector†						
Public	16.9	Ref	Ref	17.2	Ref	Ref
Private/NGO	15.1	0.87 (0.72–1.06)	1.01 (0.82–1.2)	8.2	0.43 (0.31–0.60)	0.34 (0.27 – 0.49)

Birth weight‡						
Less than 2.5 kg	27.0	2.26 (1.66–3.07)	2.22 (1.58–3.12)	27.9	2.44 (1.66–3.58)	2.41 (1.61 –3.62)
2.5–4.0 kg	14.1	Ref	Ref	13.7	Ref	Ref
More than 4.0 kg	17.0	1.25 (0.94–1.65)	1.08 (0.79–1.48)	11.7	0.84 (0.57–1.23)	0.83 (0.56 –1.23)

Parity *age interaction			1.32 (1.18–1.48)			1.16 (1.02–1.32)

* OR adjusted to the factors that were significant in bivariate analysis. Multiple regression analysis included type of delivery as the outcome variable. The independent variables were: age, area of residence, access to place of delivery (West Bank only), sector, parity, and birth weight. Significant interaction was found between parity and age, and the interaction term was included in the model.

† The public sector includes governmental hospitals and centers; the private/ngo sector includes ngo hospitals and private hospitals and clinics.

‡ Figures include low birth weight due to prematurity and birth weight that is low for gestational age.

**Table 3:** Maternal and newborn outcomes related to cesarean section deliveries.

	CS	Vaginal	*p*
% Reporting not breastfeeding at all	6.2	2.1	<0.001
Mean breastfeeding duration (months) (SE)	13.8 (0.33)	14.3 (0.12)	0.112
% Reporting visiting a physician postpartum*	57.5	20.2	< 0.001

* General practitioner or specialist.

## Footnotes

1 For more details on the MICS program, see the UNICEF website at (http://www.unicef.org/statistics/index_24302.html).
